# Association between overweight/obesity and iron deficiency anaemia among women of reproductive age: a systematic review

**DOI:** 10.1017/S1368980024001794

**Published:** 2024-09-26

**Authors:** Qonita Rachmah, Prasenjit Mondal, Hai Phung, Faruk Ahmed

**Affiliations:** 1 Public Health, School of Medicine and Dentistry, Griffith University, Gold Coast, QLD, Australia; 2 Department of Nutrition, Faculty of Public Health, Universitas Airlangga, Surabaya, Indonesia

**Keywords:** Anaemia, Hepcidin, Iron status, Obesity, Women of reproductive age

## Abstract

**Objective:**

Numerous studies have examined the relationship between overweight/obesity and iron deficiency anaemia (IDA) across diverse population groups, but a definitive link has not been clearly determined. This systematic review examined the association between overweight/obesity and IDA in women of reproductive age (WRA).

**Design:**

The initial search was performed in the CINAHL, Embase, MEDLINE, SCOPUS and Web of Science databases. The studies included should report at least one Fe status with/without an inflammatory marker, using the BMI to define overweight/obesity. Only baseline data were extracted for longitudinal studies.

**Setting:**

Global.

**Participant:**

Pregnant or non-pregnant women aged 18–50 years.

**Results:**

In total, twenty-seven papers were included (twelve addressing pregnant women and fifteen addressing non-pregnant women). Overall, most of the studies reported no association between overweight/obesity and Hb concentration. However, a positive association was reported more frequently in pregnant women. The association between overweight/obesity and serum ferritin concentrations was mixed. Most of the studies on non-pregnant women reported a positive association. Only a few studies measured hepcidin and inflammatory markers, and the majority revealed an increased level among overweight/obese WRA. Among pregnant women, overweight/obesity was positively associated with anaemia and IDA but negatively associated with iron deficiency (ID). Meanwhile, overweight/obese non-pregnant women were positively associated with anaemia, ID and IDA.

**Conclusions:**

Overweight/obesity was associated with a decreased prevalence of anaemia and IDA but an increased prevalence of ID, while its association with several Fe markers was inconclusive. Further studies integrating the assessment of various Fe markers, inflammatory markers and hepcidin are needed.

Overweight/obesity (OWT/OB) and anaemia have globally emerged as major nutritional problems in low- and middle-income countries. The prevalence of overweight/obesity is 34 %, which has doubled in the past three decades to 1·5 billion overweight/obese adults worldwide. The prevalence of overweight/obesity is higher in women than men^(1)^. An alarming trend has also been observed in the Southeast Asia region, where overweight/obesity escalated from 18·9 % in 2006 to 26·6 % in 2016^(2–4)^. Simultaneously, the latest data in 2019 reported that anaemia appears to afflict half a billion women of reproductive age (WRA, 15–49 years), affecting 32 million pregnant women and 539 million non-pregnant women. Southeast Asia is one of the most affected regions, with an estimated 244 million women being affected^(5)^. Anaemia in WRA causes fatigue and lower productivity, and more importantly, it impacts their nutritional status during pregnancy^(6,7)^. Anaemia during pregnancy is linked to adverse maternal and fetal health consequences, including premature labour, maternal mortality, low birth weight, a weakened immune system resulting in a higher burden of infectious diseases, slowed fetal and child growth and development, and neonatal anaemia^(8–11)^.

In most low- and middle-income countries, more than half of anaemia cases are iron deficiency anaemia (IDA), which is a consequence of iron deficiency (ID)^(12,13)^. Additionally, the double burden of malnutrition in terms of the co-existence of overweight/obesity and anaemia is a significant public health problem in low- and middle-income countries^(14,15)^. In susceptible population groups such as women, overweight/obesity and IDA may have a more negative impact on health than each of these disorders alone^(16)^. A study conducted with nationally representative data from fifty-two low- and middle-income countries in 2022 reported a prevalence of concurrent overweight/obesity and anaemia among 12·4 % of WRA^(17)^ Studies have reported that ID may be precipitated by overweight/obesity in non-pregnant WRA^(18)^ as well as pregnant ones^(19)^. This may be attributed to adiposity-induced low-grade inflammation due to cytokine production, which increases hepcidin synthesis and leads to a lower Fe absorption rate and lower Fe bioavailability^(20–22)^. Thus, overweight/obese individuals are more likely to have IDA compared to individuals with normal weight. However, current literature has reported contrasting results; some studies revealed that OWT/OB was associated with ID, lower serum ferritin and lower serum Fe^(23–25)^, while others did not find any association^(26–28)^. Hence, there is a need to explore the existing evidence systematically to identify the association between overweight/obesity and IDA.

Only one systematic review published in 2011 examined the relationship between overweight/obesity and Fe status. It reported a reduced TS% but increased Hb and ferritin concentrations^(29)^ in overweight/obese individuals, which is contrary to the existing evidence regarding overweight/obesity and IDA relationship^(30)^. However, the review included studies involving a wide range of population groups, including WRA, postmenopausal women, male and bariatric surgery patients. It is well known that the physiological differences between various population groups can influence the Fe biomarker levels and, thus, the reported association. Further, they failed to adjust serum ferritin concentration for inflammation and explain the hepcidin-induced inflammation among obese individuals due to a few studies that included hepcidin and inflammation markers. Filling these gaps is crucial for future studies in concluding the association between overweight/obesity and IDA, which will allow early prevention of IDA in this population. Thus, the present systematic review examined the current evidence on how overweight/obesity impacts Fe status among the specific population group of WRA.

## Methods

### Study selection

Studies that reported an association between overweight/obesity and Fe status or anaemia in pregnant and/or non-pregnant WRA were included in this systematic review. With the inclusion criteria applied, this systematic review only included studies involving pregnant women and/or non-pregnant WRA (18–50 years), reporting at least one Fe status marker (Hb, serum Fe, serum ferritin, transferrin saturation (TS%) and soluble transferrin receptor (sTfR)), with or without hepcidin or inflammation marker (C-reactive protein (CRP), alpha(1)-acid glycoprotein (AGP), IL-6 and leptin), using BMI as the criteria to define overweight/obesity, and being published in English with available full texts. In detail, BMI cut-offs are used to classify nutritional status, which is defined using WHO classification, either global or Asian classification, depending on the study population. Anaemia was defined using WHO cut-offs for pregnant (Hb <110 g/l) and non-pregnant women (Hb <120 g/l)^(12)^. Furthermore, we refrained from predefining any specific cut-off points for ID due to variations observed in different studies.

However, this systematic review did not include studies involving obese patients who had undergone bariatric surgery and participants with co-morbidities (diabetes, metabolic syndrome, hypertension and liver diseases), non-original articles (literature/systematic reviews, meta-analyses, comments, short communications and editorial letters), being unpublished and being accessible for abstract only.

### Search strategy

**Fig. 1:**
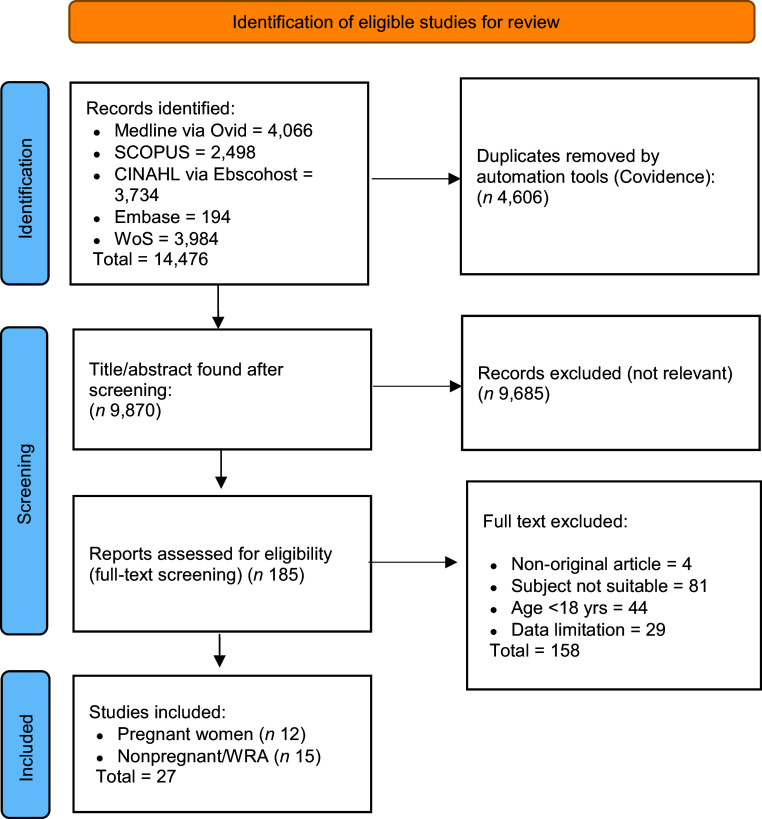
PRISMA diagram of the literature search process. PRISMA, Preferred Reporting Items for Systematic Reviews and Meta-Analyses; WRA, women of reproductive age.

A literature search was performed in the following electronic databases: SCOPUS (Griffith University Library; https://www-scopus-com.libraryproxy.griffith.edu.au/), Medline (Ovid; https://ovidsp-dc1-ovid-com.libraryproxy.griffith.edu.au/), CINAHL (EBSCO host; https://web-p-ebscohost-com.libraryproxy.griffith.edu.au/ehost/search), Embase (https://www-embase-com.libraryproxy.griffith.edu.au/search/) and Web of Science (https://www.webofscience.com/wos/woscc/basic-search). The following search terms used were (anaemia OR anaemia OR iron-deficient* OR iron status OR nutritional biomarker) and (obes* OR overweight OR BMI OR nutritional status), and (women OR women of reproductive age OR women in reproductive age OR female OR pregnant women) (see online supplementary material, Supplementary 1). To identify potential publications, we restricted our search to articles written in English, but there was no restriction on the research type. Figure 1 shows the Preferred Reporting Items for Systematic Reviews and Meta-Analyses (PRISMA) diagram of the literature search^(31)^.

### Data extraction

The next step was title/abstract screening, where we included papers meeting the eligibility criteria. This was followed by full-text screening by two independent researchers (QR and PM). Any discrepancies arising at this final screening stage were resolved through consensus building. The researchers engaged in thorough discussions to identify the root causes of discrepancies, allowed for exploring different perspectives and facilitated the alignment of interpretations. In cases where consensus could not be reached immediately, a third reviewer (FA) with expertise in the field was consulted to provide additional insights and facilitate resolution.

Data extracted from each study consisted of journal identity (title, first author, year and country of study), study methodology (study design, sample size and calculation, exposure and outcome measurements, and statistical analysis), participant characteristics (age, pregnancy status, BMI and other overweight/obesity diagnoses), and means and standard deviations (or medians and interquartile ranges) of haematological markers (Hb, MCV, MCH, MCHC and erythrocytes count), Fe markers (serum ferritin, serum Fe, serum hepcidin, TS%, sTfR and total iron-binding capacity) or inflammatory markers (CRP, IL-6, leptin and AGP) if available. In comparison to cross-sectional studies, we only extracted baseline data from the longitudinal cohort and interventional studies.

### Quality assessment of the studies

A critical appraisal of the quality of the selected research papers was performed using the Joanna Briggs Institute’s (JBI) critical appraisal tools for quantitative research^(32)^ and an additional quality checklist for epidemiological studies previously used in another systematic review^(29)^. Considering the variation of study design of included studies in this systematic review, JBI was appropriate to use as it covers the assessment of studies with a wide range of study designs and is considerably the newest validated tool for quality assessment^(33)^. The following screening questions used to check for quality: were was the study population sufficiently described?; were the sampling frame and technique sufficiently described?; how was the sample size determined?; were the participants’ inclusion and exclusion criteria appropriate?; were exposures and outcomes (anthropometric and haematological) measured using appropriate methods?; were important confounders accounted for by exclusion, data separation or statistical adjustment?; was the statistical analysis sufficiently described? For the case–control studies, an additional criterion ‘Were case and control groups correctly defined?’ was employed. Each paper was assigned one score if it met the criteria described above. The maximum score based on the quality assessment was 6. See online supplementary material, Supplementary Tables 1 (for pregnant women) and 2 (for non-pregnant women) present the final quality assessments.

## Results

### Studies included in the review

Figure 1 presents a PRISMA diagram summarising the literature search process. We used a combination of keywords to achieve a more robust search (see online supplementary material, Supplemental Fig. S1). Our initial search of four electronic databases yielded 14 476 articles involving pregnant and non-pregnant women. After removing duplicates using automation tools (Covidence; https://covidence.org/), 9870 papers remained for title and/or abstract screening. After screening the full texts by applying the inclusion and exclusion criteria, twelve original articles addressing pregnant women and fifteen original articles involving non-pregnant women were included in the analysis.

### Characteristics of the included studies

Table 1 shows the characteristics of the studies included in this systematic review. Among studies involving pregnant women, there were two cross-sectional^(23,40)^, two case–control^(34,39)^, seven prospective cohorts^(21,24,35–38,41)^ and one retrospective cohort study^(22)^. Of the twelve studies, three articles recruited participants in their early pregnancy period (*n* 3; 25 %)^(21,23,38)^, four articles examined participants in the second or third trimester^(34,36,39,41)^, four articles examined the participants throughout the pregnancy^(24,35,37,40)^ and one article recruited participants at the onset of IDA at any time point during the pregnancy^(22)^. Pre-pregnancy BMI was used to measure overweight/obesity in pregnant women, except in Abbas *et al.*’s study, which used early-pregnancy BMI^(23)^. Other three studies also reported gestational weight gain^(22,36,41)^ as an additional weight indicator during pregnancy. In terms of measured outcomes, each study reported different combinations of markers. To measure Fe status in pregnant women population, concentrations of ferritin were most frequently reported (*n* 8, 67 %)^(21,23,24,35,36,39–41)^, followed by Hb (*n* 6, 50·0 %)^(23,24,35–38)^ and serum Fe (*n* 5, 41·7 %)^(24,34,35,40,41)^. Serum transferrin receptor (sTfR) was reported in three studies along with Hb and ferritin^(35,36,41)^. Few studies reported TS% (*n* 3)^(24,34,41)^. In terms of inflammatory markers, CRP level was the most commonly used marker, which was employed in three studies^(34–36)^, followed by IL-6 in two studies^(34,35)^ and leptin in one study^(35)^. Flores-Quijano *et al.* measured IL-6 and leptin apart from CRP levels^(35)^. Hepcidin was only measured by five out of twelve reviewed studies^(24,34,35,39,41)^. Almost all studies (*n* 11, 92 %) compared the overweight/obesity group with the non-overweight/obesity group in the analysis^(21–24,34–37,39–41)^; only one study did not report a non-obese control group and rather categorised the BMI into tertiles^(38)^. Based on the methodological quality assessment, the overall study quality was moderate. Most studies (*n* 10) met at least four out of six quality indicators (see online supplementary material, Supplementary Table 1). Scores ranged from four to six, with a mean score of 4·7. One study that did not sufficiently describe the statistical analysis as a quality assessment indicator was excluded from the analysis.

**Table 1:** Characteristics of the included studies

Author, year (country)	Study design	Sample size and method	Exclusion criteria	Outcome measured	Time of measurement/age	Covariates measured in statistical analysis	Key findings
Pregnant women population							
Abbas *et al.*, 2017^(23)^ (Sudan)	Cross-sectional	423 (sampling method not determined)	Women with diabetes mellitus, thyroid disease, hypertension or any other chronic disease	Hb, SF, MCH, MCV, MCHC	Early pregnancy (<14 weeks of gestation)	None reported in the analysis	Fewer women with obesity were anaemic, a significantly higher number of women with obesity had Fe deficiency, and there was a significant negative association between serum ferritin and BMI.
Dao *et al.*, 2013^(34)^ (USA)	Case–control	Fifteen obese and fifteen non-obese (convenience sampling)	Women with pregestational diabetes, preeclampsia, autoimmune disease, acute infectious processes or pregnancy complications such as preterm premature rupture of membranes and chorioamnionitis	Serum Fe, TS, Hct, CRP, IL-6, hepcidin	Second trimester	None reported in the analysis	Maternal CRP and hepcidin were higher in the obese pregnant women group.
Flores-Quijano *et al.*, 2019^(35)^ (Mexico)	Cohort	93 (40 with obesity and 53 with an adequate weight); the sampling method was not determined	Women who did not agree to participate	Hb, SF, CRP, IL-6, Lp, sTfr, hepcidin, serum Fe, current infection	Throughout pregnancy (gestational weeks 13, 20, 27 and 34)	Model 1: total Fe intake and sTfR; for another variable, sTfR was replaced by hepcidin. Model 2: Model 1 + CRP	• Positive association of obesity with serum Fe, sTfr and hepcidin, but not ferritin. Ferritin was more correlated with the inflammatory markers (leptin) and hepcidin, possibly due to inflammation rather than showing Fe stores. • GLM results show that hepcidin and sTfR were higher in OB group, while serum Fe was lower, but, it become marginal when adjusted by CRP.
Jones *et al.*, 2016^(36)^ (China)	Cohort	1613 (sampling method not determined)	Women with chronic health problems, including diabetes (for which screening occurred between 24 and 28 weeks of pregnancy)	SF, sTfR and hsCRP, Zn protoporphyrin/heme	Early second-trimester pregnancy	Maternal age, parity, gestational weight gain, Fe supplementation, Fe status and inflammation in late pregnancy	Maternal obesity increases CRP and lowers body Fe and sTfR levels.
Liabsuetrakul *et al.*, 2011^(37)^ (Thailand)	Cohort	1192 (total sampling)	Women who had a gestational age of more than 32 weeks due to a limited time for follow-up before delivery or those who had a history of anthelminthic drug allergies	Hb, Hct	First, second and third trimester of pregnancies and at birth	Age, religion, occupation, parity and place of shower	Underweight women had an increased risk of anaemia; in contrast, women who were overweight or obese had a lower risk both by pre-pregnancy and pregnancy BMI.
Mayasari *et al.*, 2021^(24)^ (Taiwan)	Cross-sectional	1456 (sampling method not determined)	Participants aged ≤19 years, with multiple pregnancies, and with missing data on body weight or height	Hb, serum Fe, TS%, SF, hepcidin, total Fe-binding capacity, serum folate, serum vitamin B_12_	During pregnancy: first trimester: 13 or 17 weeks; second trimester: 22 or 26 weeks; and third trimester: >29 weeks)	Age, trimester, parity, education, household income, use of total supplements, and percent protein intake	• A U-shaped association between the pBMI and IDA was observed. • Serum hepcidin levels of pregnant women with obesity were significantly higher across the three trimesters compared with the other weight categories; in OB women, hepcidin level was higher in the third trimester. • The pBMI was positively correlated with the prevalence of mild nutritional deficiencies.
Mocking *et al.*, 2018^(38)^ 1) Ghana and 2) Indonesia	Cohort – secondary data analysis	1) 946 2) 433	–	Hb	1) <17 weeks of pregnancy 2) <18 weeks pregnancy	Maternal age, education level, and employment status, gravidity, second-hand smoking exposure in the Indonesian cohort; or anaemia-associated co-morbidities in a Ghanaian cohort	A higher early pregnancy BMI was associated with lower odds of anaemia.
Rahma *et al.*, 2018^(39)^ (Indonesia)	Case–control	31 pregnant women with obesity and 31 pregnant women without obesity (consecutive sampling)	Pregnant women who lose weight before pregnancy, leucocytosis and neutrophilia, history of diabetes in pregnancy	Serum hepcidin, SF	Second or third trimester	None reported	Median hepcidin and SF levels in pregnant women with obesity before pregnancy were higher than in pregnant mothers with normal weight, but both did not show a significant difference.
Scholing *et al.*, 2018^(21)^ (Amsterdam)	Cohort	4243 (total sampling)	Twin pregnancies and women with pre-existing or gestational diabetes, since these conditions can influence the nutrient status of women	Folate, SF, Fe, vitamin B_12_	Early pregnancy (interquartile range: 12–15 weeks)	Maternal age, education after primary school, smoking behaviour in early pregnancy, alcohol intake in early pregnancy, parity, nausea during pregnancy, weight gain during early pregnancy, ethnicity and use of supplementation	Pre-pregnancy obesity showed a significant association with an increase in SF levels, lower serum Fe compared with women of normal weight, but only SF that significant in restricted cubic spline modelling.
Shin *et al.* 2016^(40)^ (USA)	Cross-sectional	795 (2003–2012 NHANES dataset)	Participants reported unreliable dietary data	Serum folate, SF, serum Fe	Throughout pregnancy	Maternal age, race/ethnicity, family poverty income ratio, education, marital status, smoking status and physical activity level	Serum Fe was significantly higher in normal-weight pregnant women compared to overweight women.
Tan *et al.*, 2018^(22)^ (China)	Retrospective cohort	11 782 (2003–2012 NHANES)	Participants who were participating in any clinical trial.	Hb, SF	New-onset IDA at different gestational stages (i.e. first, second and third trimesters) during pregnancy	Maternal age, maternal race, education, local citizens, area of residence, annual family income, multiple gestations, parity, gestational week at the survey, egg intake per week, meat intake per week, smoking before pregnancy, nausea and/or vomiting during pregnancy, multivitamin supplement, Ca supplement, and multiple gestational co-morbidities.	Compared with women of normal weight, underweight women were associated with a higher risk of IDA and women who were overweight or obese had a lower risk of IDA.
Valdes *et al.*, 2015^(41)^ (Spain)	Cross-sectional	240 (sampling method not determined)	Participants who developed gestational diabetes	Serum transferrin receptor (sTFR) ferritin levels, hepcidin	24 weeks, 34 weeks and at delivery	None reported	• There was no difference in serum ferritin between obese and normal women early in pregnancy. However, levels in obese women fell and stayed low at term, whereas they recovered in women of normal weight. • Maternal sTFR levels correlated with maternal SF levels at different stages of gestation. • Maternal hepcidin levels were significantly higher in obese mothers than in controls.
Non-pregnant women population							
Adib Rad *et al.*, 2019^(42)^ (Northern Iran)	Cross-sectional	256 (115 normal weight and 141 obese) (sampling method not determined)	Women more than 35 and under 20 years of age, unwillingness to participate in the study, a known risk of anaemia other than IDA, chronic infectious and non-infectious diseases, use of Fe in the past 3 months, and at present, the use of methods of contraception such as DMPA, IUD, OCP and other hormonal methods, having polycythemia, and minor thalassemia	BMI, Hb, MCV, MCH, SF, serum Fe, TIBC	20–35 years	None reported	• No association was found between hematological characteristics and BMI.
Beard *et al.*, 1997^(43)^ (USA)	Intervention (RCT)	76 women with obesity (sampling method not determined)	Women with recent myocardial infarction, history of cerebrovascular, liver, or kidney disease, cancer, or thyroid disease. Participant with insulin-dependent and pregnancy also.	Plasma Fe Plasma thyroxine Hb, Hct, SF	Mean age 39–46 years	ANOVA test was done with diet and week as main effect factors, subject nested in a diet as a nested factor and diet x week as the interaction term	• Women receiving an 800 kcal/day (VLED) diet showed a 30 % decline in TS%, increased SF and plasma Fe, but no changes in Hb level.
Cepeda-Lopez *et al.*, 2011^(30)^ (Mexico)	Cross-sectional – secondary data analysis	621 (stratified sampling)	Subject with missing data	Hb, serum Fe, TIBC, TS% and HsCRP	18–50 years	Age, region, area, parity and Fe intake	• The prevalence of ID was significantly 1·92 times higher in women with obesity compared with normal-weight participants. • Serum Fe concentrations were lower in women with obesity than in normal-weight women. • CRP concentrations in women with obesity were four times those of their normal-weight counterparts. CRP but not Fe intake was a strong negative predictor of Fe status, independent of BMI.
Chang *et al.*, 2014^(44)^ (Taiwan)	Cross-sectional	1274 (multi-staged, stratified and clustered sampling)	Individuals with missing data for Fe biochemistry, anthropometry and 24-h dietary recall; total calorie intake ≥5000 kcal/day or ≤500 kcal/day; fat/carbohydrate (CHO) ratio <1 % and >99 %; and medical history of cancer, chronic liver diseases (e.g. hepatitis, liver fibrosis/cirrhosis) and chronic kidney diseases	Serum Fe, TIBC, TS%, SF	≥19 years	None reported	• BMI showed a protective effect on IDA; in women who were OWT/OB when compared with normal body weight; this may be attenuated in women who had an increased fat/CHO ratio. • Mean of age of women with IDA were higher in OWT women compared to normal weight.
Cheng *et al.*, 2013^(25)^ (Australia)	Cross-sectional	114 (convenience sampling)	Self-report of significant medical conditions (e.g. diabetes mellitus, disorders of the liver (including hemochromatosis) and kidney, autoimmune or metabolic diseases, and malignancy) or use of medications that may influence weight, Fe or inflammatory status; pregnancy or lactation; vegetarianism; Zn supplementation; smoking; and previous bariatric surgery	Hb, serum Fe, TS%, SF, hepcidin, CRP, sTfR	18–25 years	Model 1: use of contraception and ethnicity, presence of PCOS Model 2: Model 1 + BMI and body fat	• Multivariate modelling showed that BMI was a significant predictor of serum Fe, TS% and CRP. • Model 2 showed BMI was significantly associated with CRP but not body fat.
Eckhardt *et al.*, 2008^(45)^ (Egypt, Peru and Mexico)	Cross-sectional	11 965	Pregnant women, participants with missing data	Hb, Hct, MCV	18–49 years	Urban/rural designation, socio-economic status, education, parity and age	• In Egypt, women who were overweight had significantly lower odds of anaemia than women who were not overweight. • Similar results were found in Peru, but the difference was smaller in magnitude. • In Mexico, there were no differences in the odds of anaemia by BMI group, but there was an interaction showed that rural overweight was associated with lower anaemia. • Overweight women being protected from anaemia risk by 17 % only in women with high parity.
Fricker *et al.*, 1990^(46)^ (France)	Cross-sectional	20 women with obesity and 20 women without obesity (sampling method not determined)	Pregnant and lactating women, blood donors, and participants who had taken Fe or drugs likely to modify their Fe status during the 3 months preceding the survey	Serum Fe, TIBC TS% and SF	≥19 years	–	• Obese women consumed significantly more Fe than non-obese. • No difference in serum Fe or TIBC in both groups. • The obese group shows significantly higher Hb and SF.
Herter–Aeberl *et al.*, 2015^(18)^ (India)	Cross-sectional	146	Pregnant or giving birth in the 6 months before the study; has significant medical conditions other than obesity that might influence micronutrient status (cancer, HIV/AIDS, inflammatory bowel disease or hemochromatosis)	Hb, SF, sTfR, CRP, hepcidin, serum folate, Zn, and B_12_	18–35 years	None reported	• Hb levels were not differ between groups. SF was higher in OB compared to NW, and sTfR was higher in OWT than NW. Hepcidin was higher in both OWT and OB than NW. • Both BMI and body fat were associated with SF, sTfR, CRP and hepcidin.
Hiremath *et al.*, 2023^(47)^ (India)	Cross-sectional	688 (convenience sampling)	All those who were working and had any known co-morbid condition	Hb, erythrocytes, MCV, MCHC	>18 years	None reported	Hb was higher among women with a higher BMI (>23 kg/m^2^) but not significant, while MCHC was lower in the higher BMI group.
Hisa *et al.*, 2019^(48)^ (Japan)	Cross-sectional	10 598 (sampling method not determined)	Participants with missing data and those who were taking medications as treatment for anaemia	Hb	20–49 years	I. 20–34 years; age, BMI, smoking habits, drinking habits. II. 35–49 years; age, BMI, weight change over the past year, uterine myoma, duodenal ulcer, smoking habits, drinking habits	Among the sample of women aged 35 years and over who were overweight, there were lower odds of anaemia than in women of normal weight.
Jordaan *et al.*, 2020^(26)^ (South Africa)	Cross-sectional	134 (sampling method not determined)	–	Hb, Hct, MCV, MCH, TS%, ferritin, CRP	25–49 years	None reported	Women of reproductive age with obesity (BMI and body fat) were inversely correlated with MCV, MCH and sTfR but not with Hb or ferritin.
Karl *et al.*, 2009^(27)^ (USA)	Cohort	207 (volunteered sampling)	Underweight (BMI < 18·5 kg/m^2^) or obese (BMI > 30 kg/m^2^)	Hb, SF, TS%, sTfR, TNF-α	Mean age, 21 ± 5	Model 1: age, race, sTfR, TNF-α, BMI Model 2: Model 1 + body fat category	• OW and higher body fat women were having more ID than its counterparts. • Only SF showed a significantly higher level in women of reproductive age with obesity, but not in Hb or TS%.
Kordas *et al.*, 2013^(28)^ (Colombia)	Cross-sectional	3267 (random sampling)	Males; females aged >49 years; pregnant women; those lacking ferritin, Hb, weight or height data; and underweight women (BMI < 18·5 kg/m^2^)	Hb, ferritin, CRP	18–49 years	Age, employment, education, health self-assessment, reported doctor’s visit last year, marital status, relationship to HH, sex, age of head HH, region, total number of children, number of <5 children, current breast-feeding status.	• Women who were overweight or obese had a lower likelihood of anaemia than normal-weight women.
Nainggolan *et al.*, 2022^(49)^ (Indonesia)	Cross-sectional	11 471 (systematic random sampling)	–	Hb	19–49 years	Women’s age, education level, physical activity, consumption of fruits and vegetables, and the presence of communicable or non-communicable diseases	• Women who were overweight or obese were less likely to develop anaemia compared with women with a normal BMI, regardless of their MUAC scores.
Pita-Rodriguez *et al.*, 2023^(50)^ (Cuba)	Cross-sectional	742 (sampling method not determined)	Pregnant women, postpartum, or with a delivery time of fewer than 6 months, and women with sickle cell disease or seen in consultations for hematological disorders, women with acute illnesses, or those suffering from chronic diseases such as cancer, diabetes mellitus, arterial hypertension, hypothyroidism, hyperthyroidism, severe asthma, chronic obstructive pulmonary disease or renal insufficiency.	Hb, SF, sTfR, leucocytes, CRP, AGP, homocysteine	18–40 years	None reported	• Anaemia in women of reproductive age was associated with Fe storage deficiency and erythropoietic deficiency but not with inflammation. • Globally women who were overweight were found to be associated with inflammation, mainly with elevated CRP rather than with AGP. Adiposity and inflammation behaved similarly, with higher values for CRP than those for AGP.

AGP, alpha(1)-acid glycoprotein; OWT, overweight; OB, obesity/obese; NW, normal weight; CRP, C-reactive protein; Lp, leptin; MCH, mean corpuscular haemoglobin; MCV, mean corpuscular volume; MCHC, mean corpuscular haemoglobin concentration; sTfR, soluble transferrin receptor; SF, serum ferritin; TS%, transferrin saturation; TIBC, total iron-binding capacity; IDA, iron deficiency anaemia; HH, household; DHS, Demographic Health Survey; MUAC, mid-upper arm circumference; NPNL, non-pregnant non-lactating; VLED, very low energy diet; DMPA, depot medroxyprogesterone acetate; IUD, intra-uterine device; OCP, oral contraceptive pill.

We also found that four studies (33 %) did not consider any confounders, such as maternal characteristics, dietary intake, supplement use or the presence of infectious diseases, in their analysis, which might have affected the reported results^(23,34,39,41)^. In the studies that considered the effect of confounding variables in the statistical analysis, the most frequently considered confounders were maternal age, gestational age and socio-economic status^(21,22,24,35–38,40)^. Tan *et al.*’s study reported the most comprehensive list of identified confounders, including maternal age, race, education, local citizens, area of residence, annual family income, multiple gestations, parity, gestational age at the time of the survey, egg intake per week, meat intake per week, smoking before pregnancy, nausea and/or vomiting during pregnancy, multivitamin supplementation, Ca supplementation and multiple gestational co-morbidities^(22)^. However, their study failed to include Fe intake, birth spacing, infections and inflammatory disorders. Despite Fe supplementation being a well-known confounder, three of the twelve reviewed studies controlled this variable^(22,24,36)^.

In non-pregnant populations, fifteen studies were included^(18,25–28,30,42–50)^. The majority of the studies used cross-sectional designs (*n* 13, 86·7 %)^(18,25,26,28,42,44–51)^. Among the remaining two studies, one was an interventional study (43) and the other was a cohort study^(27)^. In terms of Fe markers, Hb level was the most frequently reported marker used in non-pregnant women (*n* 12; 80 %)^(18,25–28,42–44,46,47,50,51)^, followed by serum ferritin (*n* 9, 60·0 %)^(18,25–28,43,44,46,50)^, TS% (*n* 6, 40 %)^(25–27,43,46,51)^, serum/plasma Fe (*n* 5, 33·3 %)^(25,43,44,46,51)^ and sTfR (*n* 4, 26·7 %)^(18,25,27,50)^. For inflammatory markers, most studies used CRP level (*n* 6, 40 %)^(18,25,26,28,50,51)^, followed by AGP (*n* 1, 6·7 %)^(50)^ and TNF-α (TNF-α) (*n* 1, 6·7 %)^(27)^. Hepcidin level was only measured by two out of fifteen studies (13·3 %)^(18,25)^. Among the studies conducted on non-pregnant women, only 73 % of the studies met at least four quality assessment indicators. In addition, 40 % of the studies did not consider the effects of any potential confounders in their statistical analyses, which might have introduced significant bias while concluding the findings^(18,41,42,44,47,48)^. Studies conducted by Fricker *et al.* ^(46)^ and Nainggolan *et al.* ^(49)^ measured sex, menopausal status, oral contraceptive use, blood donation, Fe treatment/drugs, amenorrhoea, chronic disease, education level, physical activity, consumption of fruits and vegetables, and the presence of infectious or non-communicable diseases as confounders; however, both failed to account for dietary intake (Fe intake, Fe enhancers and inhibitors) and supplement use. Overall, quality scores ranged from 2 to 6, with a mean score of 4·1. Studies with a quality score of less than 2 usually did not report outcome measurements, statistical analysis or sampling procedures, and those studies were excluded from the analysis (see online supplementary material, Supplementary Table 2).

### Outcomes in pregnant women participants

Summaries of the extracted data for the pregnant women are presented in see online supplementary material, Supplementary Table 3. The prevalence of overweight/obesity among pregnant women in this review ranged from 10·8 % to 69·8 %^(21–24,36,37,40)^. In comparison, anaemia prevalence ranged from 12·7 % to 57·7 %^(23,37)^. Various Fe markers were reported in the reviewed studies, as previously mentioned. Out of six studies that measured Hb, three reported a significant positive association between overweight/obesity and Hb levels,^(24,36,38)^ while the others found no association. Three (out of five) reported a decrease in serum Fe^(21,35,40)^ among overweight/obese pregnant women, while the other studies demonstrated no association.^(34,41)^. One out of three studies showed a decrease in TS%^(24)^, while the others (*n* 2) showed no association^(34,41)^. One study (out of seven) revealed a decrease in serum ferritin^(23)^ among overweight/obese individuals, one showed an increase in serum ferritin^(24)^ and the other five studies did not report any association^(21,35,36,39,40)^. Among the seven studies that measured serum ferritin, only two measured inflammatory markers^(35,36)^. However, they did not adjust serum ferritin levels for the presence of inflammation. Lastly, one (out of three) study reported a significant increase in sTfR levels,^(36)^ while the other two did not report any difference^(35,41)^.

Only three studies measured CRP, and all of them reported an increased level in overweight/obese pregnant women^(21,34,35)^. An increase in leptin and IL-6 levels was also found by Flores-Quijano *et al.* ^(35)^. However, one study reported that there was no difference in IL-6 levels between overweight/obese and normal weight^(34)^. Moreover, four out of five studies found significantly higher hepcidin in overweight/obese pregnant women compared to normal weight^(24,34,35,41)^, while another one failed to find a significant association^(39)^. In pregnant women, overweight/obesity was found to be associated with a significantly higher proportion of ID in two studies^(23,24)^. Despite this, out of the five studies that compared the proportion of anaemia between overweight/obesity and normal weight, four reported a lower proportion of anaemia among overweight/obese women^(23,24,37,38)^, while one reported no difference^(36)^. Among three studies that measured IDA, two found a lower prevalence of IDA among overweight/obese women^(22,24)^, while the other found no association between overweight/obesity and IDA^(23)^.

A retrospective cohort study in China found that underweight women had a higher risk of IDA compared to normal-weight women. Although the risk was lower in women with obesity, the higher rate of gestational weight gain was associated with a higher risk of IDA^(22)^. Abbas *et al.* ^(23)^ in their study found a relationship between obesity and ID among pregnant women, where obese pregnant women had significantly lower serum ferritin levels (*P* = 0·010), and thus, had a higher proportion of ID (*P* = 0·015) than their counterparts. Surprisingly, the same study also reported a lower prevalence of anaemia (*P* < 0·001) in overweight/obese women without any significant difference in Hb concentration. In conclusion, overweight/obesity in pregnant women studies were generally associated with higher levels of Hb, hepcidin and inflammatory markers, and lower serum Fe, but not associated with the serum ferritin, serum TS% and sTfR. This resulted in a lower proportion of anaemia and IDA in overweight/obese pregnant women.

### Outcomes in non-pregnant women participants

The extracted data for non-pregnant women are summarised in see online supplementary material, Supplementary Table 4. Prevalence of overweight/obesity ranged from 25·3 % to 70·78 %^(18,27,28,44,45,47,48,50,51)^. Anaemia prevalence in non-pregnant women ranged from 10·0 % to 22·3 %^(25,28,44,48,49)^. Many studies used Hb as a proxy parameter to measure Fe status, and none of them demonstrated a significant decrease in Hb concentration among overweight/obese non-pregnant women. Instead, three studies reported inverse results, in which Hb levels increased among overweight/obese compared to normal-weight non-pregnant women^(25,28,46)^. Seven out of twelve studies that measured Hb did not show any association between Hb and overweight/obesity^(18,25–27,42,47,51)^, three found an increase of Hb among overweight/obese women^(28,44,46)^ and the rest (*n 2*) did not test the association^(48,50)^. Out of nine studies that measured ferritin^(18,25–28,43,44,46,50)^, six studies revealed a positive association between overweight/obesity and serum ferritin levels^(18,25,28,43,44,46)^. Among those six studies, three did not report any inflammatory marker^(43,44,46)^, while others that measured inflammatory markers did not adjust ferritin for the presence of inflammation^(18,25,28)^. Only one study adjusted ferritin levels for inflammation status, which reported lower ferritin was associated with central adiposity as indicated by waist circumference (OR = 0·59 (0·38–0·91)) but not with BMI^(50)^.

A few other studies have used serum Fe (*n* 5), TS% (*n* 6) and sTfR (*n* 4) to measure Fe status. One study reported a decrease in serum Fe concentration in overweight/obese subjects^(25)^, while two reported the opposite^(43,51)^ and two did not find any association^(44,46)^. Higher serum TS% in overweight/obese non-pregnant women was only seen in one study^(43)^ out of six, whereas two reported lower serum TS%^(25,26)^. The rest of the studies (*n* 3) did not find any difference^(27,46,51)^. Of the four studies that measured sTfR, one found an increase in sTfR among overweight/obese women^(18)^, two showed no association^(25,27)^ and one did not compare sTfR between the groups^(50)^. Herter *et al.* in their study reported a higher hepcidin level in overweight/obese non-pregnant women^(18)^, while the other study reported no association^(25)^. Higher CRP was reported among overweight/obese subjects in all six studies^(18,25,26,28,50,51)^. Six out of seven studies assessing anaemia reported a lower proportion of anaemia among obese women^(28,44,45,47–49)^, while the other reported no difference in the anaemia proportion^(50)^. ID was measured in four studies, two of which reported a lower prevalence of ID among overweight/obese women^(27,44)^, one of which reported a higher prevalence^(51)^ and one of which found no association^(28)^. IDA was reported in only one study in which a lower prevalence of IDA was found among obese women^(44)^.

In a study among non-pregnant Cuban, anaemia was associated with ID (OR: 3·02 (1·82–5·03)) and erythropoietic deficiency (sTfr >8·3 µg/ml) (OR: 5·62 (3·03–10·39)), but not with overweight (OR:0·80 (0·57–1·12)), central adiposity (OR:0·80 (0·57–1·12)) and inflammation (OR: 1·00 (0·65–1·54))^(50)^. The study also reported that overweight/obesity was correlated with elevated CRP levels (OR: 3·06 (1·89–4·94)) rather than with AGP levels (OR: 1·80 (1·05–3·08))^(50)^. In conclusion, studies among non-pregnant women revealed that overweight/obesity was generally associated with higher ferritin and serum Fe levels but not with Hb, sTfR, TS% and total iron-binding capacity concentrations. An increased level of CRP, AGP and hepcidin among obese non-pregnant women were also reported in most of the studies. Overweight/obese non-pregnant women also had a lower prevalence of anaemia and IDA, but the association with ID was mixed.

## Discussion

To the best of our knowledge, this systematic review is the first study addressing literature reporting the association of overweight/obesity with Fe status and anaemia in WRA, including pregnant women. Overall, the majority of the studies (pregnant and non-pregnant women) have demonstrated a significant inverse association between overweight/obesity and the prevalence of anaemia and IDA. On the other hand, there was a higher prevalence of ID among overweight/obese women. When looking at the individual Fe biomarkers to assess Fe status in the included studies, overweight/obesity was positively associated with the concentrations of serum ferritin, hepcidin and other inflammation markers. Still, it was negatively associated with serum Fe. Overall, no association was found between sTfR, TS% and Hb in WRA.

Although serum ferritin is the most reliable and commonly used biomarker for ID at the population level^(52)^, it is also an acute-phase protein that increases (irrespective of Fe status) in the presence of subclinical infection or inflammation, including chronic inflammation caused by excessive body fat. Of note, earlier studies have demonstrated a correlation between increased serum ferritin and CRP levels, a metric for measuring systemic inflammation, particularly during the start of an infection or inflammatory response^(18,53,54)^. Thus, higher serum ferritin among overweight/obese women, reported in this review, does not necessarily reflect an actual increase in Fe storage but could be the result of low-grade, chronic inflammation associated with overweight/obesity^(29,55,56)^. To assess Fe status using serum ferritin levels, adjustment for subclinical infection/inflammation is required using inflammatory markers such as CRP and AGP^(57,58)^. Although nine of twenty-seven primary studies measured the CRP levels, only one study adjusted the serum ferritin concentration for high CRP levels^(50)^. This study revealed that ID was not associated with being overweight but associated with central obesity as indicated by waist circumference^(50)^. Adiposity was associated with inflammation as indicated by increased CRP and AGP^(50)^. Hence, future studies exploring the association between overweight/obesity and Fe status should consider assessing Fe biomarkers along with inflammatory markers to adjust for subclinical infection or inflammation while assessing Fe status.

sTfR is an Fe-binding protein crucial for transporting Fe to the target tissues^(59)^. Thus, an increase in sTfR reflects insufficient Fe stores and indicates Fe-deficient erythropoiesis. Among six studies that examined the association between overweight/obesity and sTfR^(18,25,27,35,36,41)^, two reported a significant increase in sTfR among overweight/obese pregnant and non-pregnant women^(18,36)^. It is noteworthy that, unlike serum ferritin, sTfR remains unaffected by inflammation and thus remains a more reliable marker of Fe storage if inflammation markers are not measured^(52)^. Therefore, the studies that found overweight/obesity is associated with lower Fe storage (higher sTfR) support the known pathway of overweight/obesity and IDA. However, the majority of the studies found no association between overweight/obesity and sTfR levels. Karl *et al.* explained that a certain critical level of body fat in overweight/obese individuals may contribute to an increase in sTfR and support the overweight/obese–IDA relationship^(27)^.

Decreased serum Fe was observed among overweight/obese pregnant and non-pregnant women in four studies^(21,25,35,40)^ implying less Fe accessible^(35)^. Moreover, decreased serum Fe could be explained by the increased inflammation among the overweight/obese population. When inflammatory cytokines are released, Fe is more readily taken up and retained by reticuloendothelial system cells, preventing it from being transported to the bone marrow for erythropoiesis^(60)^. Alternatively, decreased serum Fe could also be explained by haemodilution in overweight/obese women, which can occur due to an increase in total blood volume^(61)^. During transport, Fe is bound to transferrin, and the level of transferrin bound to Fe (or TS%) can be considered as an indicator of circulating Fe^(62)^. During the second and third stages of ID, TS% decreased along with serum Fe and ferritin. Overall, only a few studies reported a lower TS% among overweight/obese WRA^(24–26)^. The pathway responsible for the decrease in TS% among overweight/obese might be attributable to impaired Fe absorption, which is consistent with persistently present low-grade inflammation and hepcidin elevation among overweight/obese individuals^(18,63)^.

Hepcidin is one of the most significant markers of Fe status, which plays a role in Fe metabolism^(64)^. Hepcidin controls the amount of Fe in the blood by binding to ferroprotein, which causes ferroprotein to be internalised and degraded and prevents cellular Fe transfer into plasma^(65)^. Ferroprotein is an Fe exporter on the surface of absorptive intestinal enterocytes, hepatocytes, macrophages and placental cells, all of which release Fe into the plasma^(65)^. Hepcidin also delays the release of recycled Fe from macrophages to the periphery and the mobilisation of Fe from the liver or spleen reserves^(66,67)^. During the second and third stages of ID, hepcidin release is decreased as a feedback mechanism, especially during erythropoiesis, to ensure Fe availability for Hb synthesis^(64)^. This is also observed during pregnancy to meet the increased Fe requirements of the mother and the fetus irrespective of overweight/obesity^(65)^. In addition, hepcidin is also produced in small amounts by adipose tissue and is higher in overweight/obese individuals^(68)^, which could also be attributed to inflammation related to overweight/obesity through the JAK/STAT pathway^(30)^. This phenomenon was also seen in all the included studies (*n* 4) that measured hepcidin as they showed an association between overweight/obesity and hepcidin^(18,24,34,35)^. This means overweight/obesity and hepcidin contribute to the mechanism during the Fe-deficient stage. Supporting this finding, a study in Taiwanese pregnant women proved that hepcidin levels were significantly higher in obese pregnant women until third trimester regardless of Fe status^(24)^. Thus, the result of this review emphasises the need for hepcidin measurement in overweight/obese and IDA association.

An association between overweight/obesity and IDA is hypothetically attributed to adiposity-induced low-grade inflammation due to cytokine production^(20)^. Elevated inflammatory cytokines such as CRP, AGP and IL-6 levels have also been reported among pregnant women with overweight/obesity compared to their counterparts^(34–36)^, as well as in overweight/obese non-pregnant women^(18,25,26,28,50,51)^. Acute-phase proteins such as CRP and AGP increase during infection or inflammation. They are often used collectively with markers of Fe status, such as serum ferritin, to help interpret the results^(59)^. CRP was the most frequently reported inflammation marker in the reviewed papers and levels aligned with obesity-related inflammation. Additionally, a relationship between CRP and ferritin in overweight/obese women found in one of the included studies^(18)^, as well as the association between IL-6 and ferritin^(35)^, strengthen the proposed pathway, which explicitly explains the role of adiposity in the relationship between inflammation and increased ferritin due to adiposity^(29)^.

Hb level is the key indicator to define anaemia, which occurs when Hb concentration is less than a specified cut-off point based on gender, age, physiological status, smoking habits and altitude^(12)^. Based on the known pathway of overweight/obese-related IDA, the Hb level should be decreased due to inhibited Fe absorption^(20,67)^. Several studies used Hb as a proxy indicator to define IDA. However, the majority of the studies failed to find an association between overweight/obesity and Hb levels; although in pregnant women, most of the studies reported a higher Hb among overweight/obese individuals^(24,36,38)^. None of the studies that reported increased Hb explained their findings. In a separate report, it was explained that obese people were more likely to have co-morbid conditions such as persistent tissue hypoxia because of sleep apnoea causing polycythaemia and increasing Hb^(29)^. Moreover, higher Hb may also be mediated by higher Fe intake in overweight/obese women^(69)^. Unfortunately, this systematic review failed to confirm the assumption since none of our included studies measured Fe intake among WRA. Menzie *et al.*, in their research, reported higher heme-iron and animal protein intake but lower vitamin C and Ca intake among obese adults than non-obese counterparts^(70)^. However, dietary differences were not associated with the inverse association between obesity and low serum Fe.

Even though most of the included studies measured Hb or ferritin as Fe status markers, very few categorised these variables into anaemia, ID or IDA and compared overweight/obesity and normal weight WRA. Overall, most of the studies that compared these parameters reported a lower prevalence of anaemia and IDA in overweight/obese WRA. On the other hand, ID was higher among overweight/obese WRA in most of the studies, including studies in pregnant women. The differences in results between different studies could be due to using different markers to define ID, such as unadjusted ferritin^(23,27,44)^, TS%^(24,27,44,51)^, serum Fe^(51)^, total iron-binding capacity^(51)^, Red Cell Distribution Width (RDW)^(27)^ or a combination of these. Additionally, confounding factors such as age, education, menstrual loss and dietary intake, as well as parity and gestational age^(71)^ in pregnant women, were not considered in many included studies. Moreover, the small sample sizes may have contributed to these inconclusive results. The degree to which overweight/obesity-related inflammation alters Fe metabolism is yet unknown; thereby, it is challenging to come to a uniform conclusion regarding the association between overweight/obesity and IDA in WRA. Measuring inflammatory markers with hepcidin and Fe status markers is crucial to understanding the relationship between overweight/obesity and IDA. However, only a few studies (four out of twenty-seven) simultaneously measured Fe status, hepcidin and inflammation markers, limiting our ability to discuss the results and draw conclusions.

Despite some limitations in this review, including a limited measure of confounding factors, dietary intake assessment and a limited number of studies that adjust ferritin for inflammation, this review has several strengths. This review included the broader use of Fe status markers, including hepcidin and various inflammation markers (CRP, AGP and IL-6), which were not addressed much in the previous systematic review. Moreover, this review also explored the association in a specific population group, such as WRA aged 18–50 years, which added to the body of knowledge in the field.

## Conclusion

Despite the limitations of the selected articles in reporting Fe status markers altogether with hepcidin and inflammatory markers to substantiate the relation between overweight/obesity and IDA, this review demonstrates that overweight/obese WRA tend to exhibit a lower prevalence of anaemia and IDA but a higher prevalence of ID. We also demonstrated overweight/obese WRA had higher hepcidin and inflammatory markers, but there was no significant overall association between Hb, sTfR and TS% with overweight/obesity. Nevertheless, this systematic review captures the lack of well-designed studies to explain the association between overweight/obesity and IDA among WRA, including relatively few longitudinal studies. Our review underscores the urgent need for further studies to be carried out more comprehensively, considering a set of Fe markers, hepcidin and inflammation markers, as well as potential confounders, particularly dietary Fe intake or Fe supplementation, that could highly influence the association between overweight/obesity and IDA.

## Supporting information

Rachmah et al. supplementary material Rachmah et al. supplementary material

## Supplementary material

For supplementary material accompanying this paper visit https://doi.org/10.1017/S1368980024001794
