# Supplementary material for: Association between overweight/obesity and iron deficiency anaemia among women of reproductive age: a systematic review
Source: Public Health Nutr. 2024 Sep 26;27(1):e176. doi: 10.1017/S1368980024001794 (PMC11504706; doi:10.1017/S1368980024001794)
Supplement: Rachmah et al. supplementary material [file S1368980024001794sup001.doc]

1. MeSH – Anemia, Iron-deficiency, Iron status
2. Keyword – Anemi*
3. Keyword – Anaemi*
4. Keyword – Iron Deficien*
5. Keyword – Iron status
6. Keyword – Nutritional biomarker
7. MeSH – Obesity; Overweight; Body Mass Index
8. Keyword – Obes*
9. Keyword – Overweight
10. Keyword – Body Mass Index
11. Keyword – Nutritional status
12. MeSH – Women; Female; Pregnant women
13. Keyword – Women
14. Keyword – Female
15. Keyword – Women in reproductive age
16. Keyword – Women of reproductive age
17. Keyword – Pregnant women
18. 2 OR 3 OR 4 OR 5 OR 6
19. 8 OR 9 OR 10 OR 11
20. 13 OR 14 OR 15 OR 16 OR 17
21. 18 AND 19 AND 20

**Supplementary Figure 1.** Electronic search strategy used to search the database; modified to comply with search rules of each database. Limited search to article in English and journal articles.

*asterisk denotes the use of truncation

**Supplementary Table 1. Quality item checklist (pregnant women population)**

| **Author, year**  **(country)** | **Study population sufficiently described? (age, pregnancy age)** | **Sampling procedures sufficiently described? (sampling frame, sample size, group allocation)** | **Appropriate participant inclusion and exclusion criteria?** | **Exposure and outcomes collected using appropriate methods? (anthropometry, blood sample collection)** | **Identified confounder (maternal age, gestational age, education, ethnicity, area of residence, income, dietary intake, alcohol consumption, supplement use, hemodilution, number of parities, birth spacing, acute infection, inflammatory disorders, chronic disease)** | **Statistical analysis sufficiently described?** |
| --- | --- | --- | --- | --- | --- | --- |
|  |  |  |  |  |  |  |
| Abbas et al., 2017 (Sudan) | Yes | No (sampling method not reported) | Yes | Yes | None reported in analysis | Yes |
| Dao et al., 2013 (USA) | Yes | No (sample size determination not reported) | Yes | Yes | None reported in analysis | Yes |
| Flores-Quijano et al., 2019 (Mexico) | Yes | Yes | Only inclusion criteria | Yes | Yes (cofounders accounted for: the existence of an underlying health condition, gestational age, presence of infection, and the development of any complication) | Yes |
| Jones et al., 2016 (China) | Yes | No (sample size determination not reported) | No | Yes | Yes (confounders accounted for: maternal age, parity, gestational weight gain, iron supplementation, iron status and inflammation in late pregnancy, cord hsCRP concentration) | Yes |
| Liabsuetrakul et al., 2011 (Thailand) | Yes | No (sample size determination not reported) | No | No | Yes (confounders accounted for: age, religion, occupation, parity, and place of shower) | Yes |
| Mayasari et al., 2021 (Taiwan) | Yes | No (sample size determination not reported) | Yes | Yes | Yes (confounders accounted for: age, trimester, parity, education, household income, use of total supplements and % protein intake) | Yes |
| Mocking et al., 2018 (Ghana and Indonesia) | Yes | No (sample size determination not reported) | No | No | Yes (confounders accounted for: maternal age, education level and employment status, gravidity, secondhand smoking exposure in Indonesian cohort or anaemia-associated-comorbidities in Ghanaian cohor) | Yes |
| Rahma et al., 2018  (Indonesia) | Yes | No (sample size determination not reported) | Yes | Yes | None reported | Yes |
| Scholing et al., 2018 (Amsterdam) | Yes | No (sample size determination not reported) | Yes | Yes | Yes (confounders accounted for: maternal age, education after primary school, smoking  behaviour in early pregnancy, alcohol intake in early pregnancy, parity, nausea during pregnancy, weight gain during early pregnancy and ethnicity) | Yes |
| Shin et al. 2016 (USA) | Yes | No (sample size determination not reported) | Yes | Yes | Yes (confounders accounted for: maternal age, race/ethnicity, family poverty income ratio, education, marital status, smoking status, and physical activity level) | Yes |
|  |  |  |  |  |  |  |
| **Author, year**  **(country)** | **Study population sufficiently described? (age, pregnancy age)** | **Sampling procedures sufficiently described? (sampling frame, sample size, group allocation)** | **Appropriate participant inclusion and exclusion criteria?** | **Exposure and outcomes collected using appropriate methods? (anthropometry, blood sample collection)** | **Identified confounder (maternal age, gestational age, education, ethnicity, area of residence, income, dietary intake, alcohol consumption, supplement use, hemodilution, number of parities, birth spacing, acute infection, inflammatory disorders, chronic disease)** | **Statistical analysis sufficiently described?** |
| Tan et al., 2018 (China) | Yes | Yes | Yes | No (blood sample measurement was not described) | Yes (confounders accounted for: maternal age,  maternal race, education, local citizens, area of residence, annual family income, multiple gestations, parity, gestational week at the survey, egg intake per week, meat intake per week, smoking before pregnancy, nausea and/or vomiting during pregnancy, multivitamins supplement, calcium supplement, and multiple gestational comorbidities) | Yes |
|  |  |  |  |  |  |  |
| Valdes et al., 2015 (Spain) | Yes | No (sample size determination & method not reported) | Yes | Yes | None reported | Yes |
|  |  |  |  |  |  |  |
|  |  |  |  |  |  |  |

**Supplementary Table 2. Quality item checklist (non-pregnant women population)**

| **Author, year**  **(country)** | **Study population sufficiently described? (age, gender, menopausal status)** | **Sampling procedures sufficiently described? (sampling frame, sample size, group allocation)** | **Appropriate participant inclusion and exclusion criteria?** | **Exposure and outcomes collected using appropriate methods? (anthropometry, blood sample collection)** | **Identified confounder (age/menopausal status, ethnicity, dietary iron intake, oral contraceptive use, blood donation, iron treatment, alcohol consumption, menorrhagia or amenorrhoea, haemorrhage or gastrointestinal bleeding, acute infection, liver disease or haemochromatosis, inflammatory disorders, chronic disease)** | **Statistical analysis sufficiently described?** |
| --- | --- | --- | --- | --- | --- | --- |
|  |  |  |  |  |  |  |
| Adib Rad et al., 2019  (Northern Iran) | Yes | No (sample size determination not reported) | Yes | Yes | None reported in analysis (subjects with possible confounding were excluded i.e. chronic infection, use of iron, contraception use) | Yes |
| Beard et al., 1997  (USA) | No (menopausal status not reported) | No (sample size determination not reported) | Yes | Yes | Yes (confounders accounted for: gender, liver disease, chronic disease (insulin-dependent diabetes mellitus) | Yes |
| Cepeda-Lopez et al, 2011 (Mexico) | Yes | Yes | No | Yes | Yes (confounder accounted for: age, region, area, and parity) | Yes |
| Chang et al., 2014  (Taiwan) | Yes | Yes | Yes | Yes | None reported | Yes |
| Cheng et al., 2013  (Australia) | Yes | No (sample size determination not reported) | Yes | Yes | Yes (use of contraception and ethnicity) | Yes |
| Eckhardt et al., 2008 (Egypt, Peru and Mexico) | Yes | No (sample size determination not reported) | Yes | Yes | Yes (confounders accounted for: urban/rural designation, socioeconomic status, education, parity, and age) | Yes |
| Fricker *et al*, 1990  (France) | Yes | No (sample size determination not reported) | Yes | Yes | Yes (confounders accounted for: gender, menopausal status, oral contraceptive use, blood donation, iron treatment/drugs, amenorrhoea, chronic disease) | Yes |
| Herter‑Aeberl et al., 2015 (India) | Yes | No (sample size determination not reported) | Yes | Yes | None reported | Yes |
| Hiremath et al., 2023  (India) | Yes | No (sample size determination not reported) | Yes | No (blood iron status analysis did not describe) | None reported | Yes |
| Hisa et al., 2019  (Japan) | Yes | No (sample size determination not reported) | Yes | Yes | None reported | Yes |
|  |  |  |  |  |  |  |
| Jordaan et al., 2020  (South Africa) | Yes | No (sample size determination not reported) | No | Yes | None reported | Yes |
| Karl et al., 2009  (USA) | Yes | No (sample size determination not reported) | No | No | None reported | Yes |
| Kordas et al., 2013  (Colombia) | Yes | Yes | Yes | Yes | None reported | Yes |
| **Author, year**  **(country)** | **Study population sufficiently described? (age, gender, menopausal status)** | **Sampling procedures sufficiently described? (sampling frame, sample size, group allocation)** | **Appropriate participant inclusion and exclusion criteria?** | **Exposure and outcomes collected using appropriate methods? (anthropometry, blood sample collection)** | **Identified confounder (age/menopausal status, ethnicity, dietary iron intake, oral contraceptive use, blood donation, iron treatment, alcohol consumption, menorrhagia or amenorrhoea, haemorrhage or gastrointestinal bleeding, acute infection, liver disease or haemochromatosis, inflammatory disorders, chronic disease)** | **Statistical analysis sufficiently described?** |
| Nainggolan et al., 2022  (Indonesia) | Yes | No (sample size determination not reported) | No | No (haemoglobin analysis by capillary blood using Hemocue  Hb 201+) | Yes (confounders accounted for: women’s age, education level, physical activity, consumption of fruits and vegetables, and the presence of communicable or non-communicable diseases) | Yes |
| Pita-Rodriguez et al., 2023 (Cuba) | Yes | No (sample size determination not reported) | Yes | Yes | None reported | Yes |

**Supplementary Table 3. Summary of outcomes for pregnant women studies**

| **Author, year**  **(Country)** | **Strata/Group** | **Age (years)** | **Pre-pregnancy BMI (kg/m^2^)** | **Haemoglobin (g/dL)** | **Ferritin (µg/L)** | **Hepcidin (µg/mL)** | **Others** |
| --- | --- | --- | --- | --- | --- | --- | --- |
|  |  |  |  |  |  |  |  |
| Abbas et al., 2017 (Sudan) | Underweight  Normal weight  Overweight  Obese | 19.5 + 6.3^a^  24.9 + 6.5  26.7 + 6.1  26.4 + 5.4 | N/A | 11.1 + 1 .2  10.4 + 0.8  10.5 + 0.8  10.5 + 0.8 | 89.5 (36.0–89.5)^a,b^  39.0 (21.0–78.0)  28.6 (15.9–53.6)  24.0 (12.3–58.0) |  | Significantly higher iron deficiency in overweight obese (p<0.05) but fewer had anaemia and no different in iron deficiency anaemia |
| Dao et al., 2013 (USA) | Obese  Lean | 30.0±3.9  32.1±5.8 | 38.6±7.0^a^  22.8±1.5 | N/A | **CRP (mg/L)**  14.3 (11.5)^a^  5.0 (4.4) | 13.5±9.0^a^  5.1±2.7 | Obese group significantly have higher hepcidin, CRP, and ratio of serum oxidized to reduced glutathione; but no difference in serum iron, Tstat, and IL-6 |
| Flores-Quijano et al., 2019 (Mexico) – 1^st^ visit wk 13 | Adequate weight  Obese (pgBMI>30kg/m^2^)  Adequate weight  Obese (pgBMI>30kg/m^2^) | 31.68 ± 5.66  31.13 ± 5.89  **sTfr (mg/L)**  13.34 (11.30–16.08)^b^  13.98 (11.03–19.30)^b^ | 22.71 ± 1.95 ^a^  31.68 ± 5.66  **IL-6 (pg/mL)**  1.79 (1.63, 2.10)^a,b^  2.15 (1.81–2.43)^b^ | 13.55 (13.18, 14.45)^b^  13.39 (13.08, 13.99)^b^  **Leptin (pg/mL)**  21.50 (15.11–26.25)^a,b^  44.48 (32.14–61.57)^b^ | 39.30 (27.60, 65.05)^b^  40.60 (19.40, 96.15)^b^  **CRP (mg/L)**  4.36 (3.04–8.58) ^a,b^  10.65 (6.84–15.40)^b^ | 8.04 (5.88, 11.86)^b^  9.58 (6.21, 15.67)^b^  **Serum iron (µg/dL)**  162.45 (129.8–199.5)^a,b^  149.76 (113.6–199.7)^b^ | - |
| Tan et al., 2018 (China) | Underweight (n, %)  Normal weight  Overweight  Obese  Rate of GWG (kg/week) | **IDA**  765 (19.54)  2805 (71.65)  321 (8.20)  24 (0.61)  0.35 (0.27–0.44) | **Non-IDA**  1263 (16.05)  5671 (72.09)  812 (10.32)  121 (1.54)  0.31 (0.21–0.42) | **OR (95% CI)**  1.22 (1.11–1.35)^a^  Ref  0.80 (0.70–0.92)^a^  0.40 (0.26–0.62)^a^  4.47 (3.56–5.62)^a^ | - | - | Lower prepregnancy BMI was associated with higher risk of IDA, and pregnant women with faster GWG may be more likely to develop IDA. |
|  |  |  |  |  |  |  |  |
| Jones et al., 2016 (China) | Underweight  Normal weight  Overweight  Obese | 24 (3.0)^a^  25 (3.5)  26 (4.3)  25 (3.6) | **sTfR**  14 (4.5)^a^  15 (4.7)  16 (5.3)  17 (6.2) | 120 (9.8)^a^  122 (9.2)  124 (8.5)  124 (8.7) | 43 (36)  41 (35)  44 (36)  37 (28) | **Body iron (mg/kg)**  5.7 (3.5)  5.2 (3.5)  5.3 (3.4)  4.7 (3.3) | **CRP (mg/L)**  2.6 (4.6) ^a^  3.4 (5.3)  5.6 (5.7)  5.7 (7.3) |
|  |  |  |  |  |  |  |  |
| Mayasari, et al. 2021 (Taiwan) | Underweight  Normal weight  Overweight  Obese | 31.6 ± 4.6^a^  32.6 ± 4.6  32.8 ± 4.8  33.0 ± 5.0 | 17.7 ± 0.6^a^  21.1 ± 1.5  25.3 ± 0.9  30.3 ± 3.1 | 11.4 ± 1.7^a^  11.7 ± 1.9  12.1 ± 2.2  11.7 ± 1.9^a^ | 11.6 (7.6-26.3)^a,b^  13.1 (7.9-27.5)  15.1 (8.88-31.5)  14.8 (7.8-32.4) | 20.1 ± 31.0^a^  23.3 ± 31.6^a^  26.1 ± 35.0^a^  27.7 ± 34.0 | **TS(%)**  17.8 ± 12.7 ^a^  16.9 ± 10.2 ^a^  16.6 ± 8.4 ^a^  13.7 ± 7.5 ^a^ |
| **Author, year**  **(Country)** | **Strata/Group** | **Age (years)** | **Pre-pregnancy BMI (kg/m^2^)** | **Haemoglobin (g/dL)** | **Ferritin (µg/L)** | **Hepcidin (µg/mL)** | **Others** |
|  |  |  |  |  |  |  |  |
| Mocking et al., 2018  (Ghana and Indonesia) | **Indonesia**  Lower tertile  Middle tertile  Upper tertile | 26.5 (6.06) ^a^  29.1 (5.18)  30.2 (6.06) | 12.5 (20.6)  20.7 (24.3)  24.4 (39.2) | **Ghana**  Lower tertile  Middle tertile  Upper tertile | **Age**  26.3 (4.5)^a^  28.0 (5.3)  29.7 (5.0) | **BMI**  15.4 (23.1)  23.1 (27.0)  27.1 (42.3) | **Linear regression results**   - Indonesia (OR 0.054; 95% CI 0.03 – 0.08)^a^ - Ghana (OR 0.044; 95% CI 0.02-0.07)^a^ |
|  |  |  |  |  |  |  |  |
| Rahma et al., 2018  (Indonesia) | Obese  Normal weight | N/A | N/A | N/A | 28.04 ± 26.69  28.76 ± 28.07 | 9.83 ± 13.21  9.58 ± 15.35 |  |
| Scholing et al., 2018 (Amsterdam) | Underweight  Normal weight  Overweight  Obese | 29.4 + 5.1^a^  31.1 + 4.7  30.6 + 5.1  31.0 + 5.5 | 17.7 ± 0.76  21.6 ± 1.7  27.0 ± 1.4  33.8 ± 4.0 | **Fe (μmol/l)**  21.9 ± 8.0^a^  22.7 ± 7.2  19.9 ± 6.7  17.6 ± 6.9  **Iron (ug/dL)** | 34.8 (23.0-60.4)^b^  42.6 (25.7-68.9)  38.1 (23.0-61.7)  43.1 (23.0-76.0) | **CRP (mg/L)**  2.0 (0.6-4.4)  2.8 (1.2-5.2)  5.8 (2.8-9.2)^a^   - 1. (5.0-14.4)^a^ | - Pre-pregnancy obesity showed a significant association (9 %; 95 % CI 6, 32 %) with increase of ferritin levels compared with women with normal weight. - Women with overweight and obesity had significantly higher odds for serum Fe deficiency (OR=1·45; 95 % CI 1·02, 2·07 and OR=3·26; 95 % CI 2·09, 5·08, respectively) |
| Shin et al. 2016 (USA) | Underweight  Normal weight  Overweight  Obese | N/A | N/A | 79.4 (9.4)^a^  86.2 (5.0)^a^  68.9 (3.0)^a^  72.2 (5.5)^a^ | 44.5 (9.2)  34.7 (3.8)  35.1 (3.7)  44.5 (6.4) | N/A | - |
| Liabsuetrakul et al., 2011 (Thailand) | Pre-pregnancy  1^st^ trimester  2^nd^ trimester  3^rd^ trimester  At birth | N/A | 21.8 (4.0)^a^  22.4 (4.1)  23.5 (4.1)  25.8 (3.8)  26.7 (4.0) | -  12.2 (1.1)  11.3 (1.2)  11.7 (1.5)  12.2 (1.4) | N/A | N/A | - Underweight women had an increased risk of anaemia (AOR 1.5; 95% CI 1.0-2.1); in contrast, the overweight and obese women had a lower risk both by pre-pregnancy and pregnancy BMI (AOR 0.7; 95% CI 0.5-.09 vs AOR 0.5; 95% CI 0.3-0.9, respectively). |
| Valdes et al., 2015 (Spain) | Control  Overweight  Obese  Control  Overweight  Obese | 30.79±4.31^a^  31.75±4.52^a^  28.98± 4.56^a^  **Tsat (%)**  **17.87±7.96^a^**  15.39±5.56  15.72±6.15 | 21.97 (20.55, 23.30)^a^  27.18 (26.56, 28.55)^a^  32.37 (31.29, 34.64)^a^  **sTfR (nmol/L)**  17.62±5.02  17.89±5.9  20.23±6.16 | 12.56±1.61  12.96±1.99  12.12±1.21 | N/A | 19.45±10.96^a^  18.26±11.17  24.96± 13.04 |  |

Data presented as mean ± SD unless otherwise specified.

BMI, body mass index; Tsat/TS,transferrin saturation ;CRP, c reactive protein; IL6, interleukin-6; IDA, iron deficiency anaemia; GWG, gestational weight gain; N/A, not applicable

^a^Statistically significant difference between group

^b^Median interquartile range

**Supplementary Table 4. Summary of outcomes for non-pregnant women studies**

| **Author, year**  **(Country)** | **Strata/Group** | **Age (years)** | **BMI (kg/m^2^)** | **Haemoglobin (g/dL)** | **Ferritin (µg/L)** | **Hepcidin (µg/mL)** | **Others** |
| --- | --- | --- | --- | --- | --- | --- | --- |
|  |  |  |  |  |  |  |  |
| Adib Rad et al., 2019  (Northern Iran) | Normal weight  Obese | - | - | 13.48±2.43  13.62±1.98 | - | - | No statistical significant found in another blood biomarkers (Hct, MCV, MCH, and RBC) |
| Beard et al., 1997  (USA) | VLED 1758 kJ/day  VLED 2763 kJ/day  VLED 3349 kJ/day | 41 ± 37.4  39 ± 37.1  46 ± 22.6 | - | 38.1 ± 7.8 | 40.1 ± 25.0  26.7 ± 16.5  32.0 ± 23.1 | **TS (%)**  18.0 ± 7.5  22.3 ± 9.1  20.0 ± 4.5 | - |
| Cepeda-Lopez et al, 2011 (Mexico) | Normal weight  Overweight  Obese  Normal weight  Overweight  Obese | 29.1 ± 8.1  31.9 ± 7.7^a^  34.9 ± 7.2^a^  **TIBC (µg/dL)**  386 ± 64  388 ± 69  397 ± 80 | 22.5 ± 1.6  27.3 ± 1.4 ^a^  33.3 ± 2.7 ^a^  **TS (%)**  18.8 ± 10.0  19.3 ± 9.6  16.7 ± 8.7 | 13.5 ± 1.6  13.5 ± 1.6  13.7 ± 1.6  **Iron def anemia (%)^c^**  48.3  52.7^a^  61.2^a^ | -  **HsCRP (mg/dL)**  1.17 (2.67)^b^  2.12 (3.47)^a^  4.44 (5.36)^a^ | - | Calcium intake (mg/day) was found to be significantly lower in obese women than normal weight. |
| Chang et al., 2014  (Taiwan) | Underweight  Normal weight  Overweight  Obese | 39.3 (1.9)^a^  49.9 (0.8)  57.2 (0.9)  56.4 (0.9) | 17.8 (0.1)  21.6 (0.06)  25.4 (0.04)  30.1 (0.1) | 12.5 (0.1)^a^  12.6 (0.1)  12.9 (0.1)  13.0 (0.1) | 55.4 (5.1)^a^  89.2 (3.6)  120.6 (5.7)  120.4 (5.6) | **Iron def anemia (%)^d^**  10.6^a^  8.2  3.2  4.3 | Negative relationships between BMI and prevalence of iron deficiency and IDA were found (p = 0.04 and p = 0.002) |
| Cheng et al., 2013  (Australia) | BMI 27.5–29.9  BMI 30.0–34.9  BMI >35.0  BMI 27.5–29.9  BMI 30.0–34.9  BMI >35.0 | 22.0±2.1  21.7±2.3  23.1±2.3  **Serum iron (mmol/l)**  17.2±6.3  16.0±7.4  13.0±6.0^a^ | -  **Tsat (%)**  25.9±11.3  22.9±10.9 19.3±9.1^a^ | 13.2±0.8  13.0±1.0  13.0±0.8  **CRP (mg/L)**  1.62 (4.15)  1.62 (4.15)^a^  6.24 (8.17)^a^ | 31.0 (34.0)  30.5 (31.0)  46.0 (49.0)^a^  **sTfR-F (µg/L)**  1.64±0.47  1.61±0.49  1.58±0.35 | 5.25 (8.28)  6.30 (7.70)  9.20 (9.78) |  |
| Eckhardt et al., 2008  **Odds ratios (CI)** | Egypt  Peru  Mexico | 0.78 (0.68, 0.90)^a^  0.83 (0.71, 0.96)^a^  0.90 (0.79, 1.03) | - | - | - | - | OVWT women had significantly lower odds of anaemia than non-OVWT women (OR¼0.78, 95% CI: 0.68, 0.90). |
| Fricker *et al*, 1990^27^  (France) | Non-obese  Obese  Non-obese  Obese | 30.5 ± 7.0  30.8 ± 9.3  **Hct**  0.39 ± 0.03^a^  0.41 ± 0.02 | 20.7 ± 1 .4  3 1.9 ± 4.0  **Serum iron (mmol/l)**  16.0 ± 5.2  14.4 ± 6.0 | 12.9 ± 1.0 ^a^  13.7 ± 0.9  **TIBC (µg/dL)**  81.5 ± 13.6  82.0 ± 9.1 | 25.8 ± 19.5^a^  48.0 ± 44.3  **TS (%)**  0.20 ± 0.07  0.18 ± 0.08 | - |  |
| **Author, year**  **(Country)** | **Strata/Group** | **Age (years)** | **BMI (kg/m^2^)** | **Haemoglobin (g/dL)** | **Ferritin (µg/L)** | **Hepcidin (µg/mL)** | **Others** |
| Herter‑Aeberl et al., 2015 (India) | Normal weight  Overweight  Obese  Normal weight  Overweight  Obese | 25.1 ± 4.4^a^  25.1 ± 4.4  26.3 ± 4.6  **sTfR (mg/dl)**  2.01 (1.68, 2.30)^a^  2.68 (2.31, 3.10)  2.49 (1.93, 3.15) | 26.3 ± 4.6^a^  27.0 ± 1.7  32.6 ± 2.4  **CRP (mg/dl)**  0.05 (0–0.86)^a^  0.20 (0–1.53)  0.41 (0–1.61) | 12.2 ± 2.4  12.5 ± 1.7  12.8 ± 1.1 | 15.0 (11.7, 19.2)^a^  22.0 (17.1, 28.4)  29.3 (20.9, 41.0) | 0.73 (0–53.9)^a^  4.43 (0.02–20.46)  6.48 (0.46–26.08) |  |
|  |  |  |  |  |  |  |  |
| Hiremath et al., 2023  (India) | BMI <23 kg/m^2^  BMI >23 kg/m^2^ | 28.26 ± 4.812^a^  31.51 ± 5.169 | - | 12.4 ± 1.17^a^  12.6 ± 1.03 | - | - | RBC and MCV did not significantly different, but MCHC was lower in higher BMI group |
| Hisa et al., 2019  (Japan) | BMI <18.5  BMI 18.5–24.9  BMI 25.0–29.9  BMI >30.0 | **Anaemia**  48 (18.3)  201 (76.4)  13 (4.9)  1 (0.4) | **Non-anaemic**  354 (21.7)  1,174 (72.2)  83 (5.1)  15 (0.9) | - | - | - |  |
| Jordaan et al., 2020  (South Africa) | Underweight  Normal weight  Overweight  Obese class 1  Obese class 2  Obese class 3 | - | -  **STfR (nmol/L)** | 13.5 (13.0, 14.6)^e^  14.4 (13.5, 14.7)  14.1 (13.1, 15.0)  13.6 (12.8, 14.1)  13.6 (12.8, 14.1)  13.8 (13.2, 14.4) | 127.0 (56.0, 134.0)^e^  179.5 (86.5, 666.5)  179.5 (86.5, 666.5)  76.5 (36.5, 163.3)  89.0 (54.5, 165.5)  49.0 (28.3, 215.0) | **TS (%)**  42.0 (27.0, 42.0)^e,a^  31.9 (23.7, 50.6)  28.9 (24.5, 35.1  28.9 (24.5, 35.1  28.5 (15.5, 34.7)  28.5 (15.5, 34.7)  **TS (%)** | - |
| Karl et al., 2009  (USA) | Normal weight  Overweight  Normal fat  Overfat | 20 ± 4^a^  22 ± 5  21 ± 5^a^  21 ± 4 | 21.9 ± 7.8  20.7 ± 7.9  21.3 ± 7.8  21.7 ± 7.8 | 12.3 ± 1.1  12.3 ± 1.1  12.3 ± 1.1  12.3 ± 1.1 | 37.9 ± 30.2  43.5 ± 30.3  37.3 ± 29.8^a^  45.8 ± 29.9 | 18.3 ± 9.5  20.1 ± 9.5  18.4 ± 9.4  20.1± 9.4 | - |
| Kordas et al., 2013  (Colombia) | 18.5–24.9 kg/m^2^  25–29.9 kg/m^2^  >30 kg/m^2^ | - | 22.1 (22.0, 22.2)^f^  27.2 (27.2, 27.3)  33.9 (33.5, 34.3) | 123 (122, 124)  125 (123, 126)^a^  124 (123, 126) | 35.1 (33.2, 37.0)  41.8 (39.1, 44.6)^a^  52.8 (46.7, 59.0)^a^ | **CRP (g/L)**  0.002 (0.002, 0.002)  0.003 (0.00, 0.003)^a^  0.005 (0.004, 0.005)^a^ | - |
| Nainggolan et al., 2022  (Indonesia) | - Women with overweight and obesity were less likely to develop anemia compared to women with a normal BMI, regardless of their MUAC scores.  - Women who had been diagnosed by health workers with a noncommunicable disease had significantly reduced likelihood of developing anemia compared to women without any such illnesses (OR = 0.75; 95%CI: 0.67–0.83). | | | | | | |
|  |  |  |  |  |  |  |  |
|  |  | | | | | | |
| **Author, year**  **(Country)** | **Strata/Group** | **Age (years)** | **BMI (kg/m^2^)** | **Haemoglobin (g/dL)** | **Ferritin (µg/L)** | **Hepcidin (µg/mL)** | **Others** |
|  |  | | | | | | |
| Pita-Rodriguez et al., 2023 (Cuba) | Data not shown in table but descriptively:   - Anemia was associated with iron storage deficiency (OR = 3.02 (1.82–5.03)) and with erythropoietic deficiency (OR = 5.62 (3.03–10.39)), but not with inflammation (OR = 1.00 (0.65–1.54)), global overweight (OR = 0.80(0.57–1.12)), and central adiposity (OR = 0.80 (0.57–1.12)). - Global overweight in women was found to be associated with inflammation (OR = 2.23 (1.41–3.53)), mainly with elevated CRP (OR = 3.06 (1.89–4.94)) rather than with AGP (OR = 1.80 (1.05–3.08)). - Adiposity and inflammation behaved in a similar way, with (OR = 3.23 (2.32–4.51)), with higher values for CRP (OR = 3.76 (2.29–6.16)) than those for AGP (OR = 1.94 (1.19–3.15)) | | | | | | |
|  |  | | | | | | |

Data presented as mean ± SD unless otherwise specified

BMI, body mass index; Tsat/TS,transferrin saturation; sTfR-F, soluble transferrin receptor-ferritin index; CRP, c reactive protein; IL6, interleukin-6; IDA, iron deficiency anaemia; TIBC, total iron binding capacity; N/A, not applicable

^a^Statistically significant difference between group

^b^Median interquartile range

^c^Iron deficiency was defined as either a low serum iron or an elevated TIBC and a low TS.

^d^Iron deficiency anemia (IDA): SF ˂12 ng/mL, TS < 15% and Hb < 12 g/dL

^e^Median (25th, 75th)

^f^Mean (95% CI)

^g^Geometric mean (95% confidence interval)
